# Strength Development Monitoring of Cemented Paste Backfill Using Guided Waves

**DOI:** 10.3390/s21248499

**Published:** 2021-12-20

**Authors:** Wen He, Changsong Zheng, Shenhai Li, Wenfang Shi, Kui Zhao

**Affiliations:** 1 School of Resources and Environmental Engineering, Jiangxi University of Science and Technology, Ganzhou 341000, China; zhengcs666@163.com (C.Z.); jxust-lsh1995@163.com (S.L.); wenfangshi@126.com (W.S.); yglmf_zk@163.com (K.Z.); 2 Jiangxi Provincial Key Laboratory of Mining Engineering, Jiangxi University of Science and Technology, Ganzhou 341000, China; 3 Engineering Research Center for High-Efficiency Development and Application Technology of Tungsten Resources, Jiangxi University of Science and Technology, Ministry of Education, Ganzhou 341000, China

**Keywords:** cemented paste backfill, guided wave, guided wave velocity, attenuation of guided wave

## Abstract

The strength of cemented paste backfill (CPB) directly affects mining safety and progress. At present, in-situ backfill strength is obtained by conducting uniaxial compression tests on backfill core samples. At the same time, it is time-consuming, and the integrity of samples cannot be guaranteed. Therefore guided wave technique as a nondestructive inspection method is proposed for the strength development monitoring of cemented paste backfill. In this paper, the acoustic parameters of guided wave propagation in the different cement-tailings ratios (1:4, 1:8) and different curing times (within 42 d) of CPBs were measured. Combined with the uniaxial compression strength of CPB, relationships between CPB strength and the guided wave acoustic parameters were established. Results indicate that with the increase of backfill curing time, the guided wave velocity decreases sharply at first; on the contrary, attenuation of guided waves increases dramatically. Finally, both velocity and attenuation tend to be stable. When the CPB strength increases with curing time, guided wave velocity shows an exponentially decreasing trend, while the guided wave attenuation shows an exponentially increasing trend with the increase of the CPB strength. Based on the relationship curves between CPB strength and guided wave velocity and attenuation, the guided wave technique in monitoring the strength development of CPB proves feasible.

## 1. Introduction

The utilization of mineral resources has extensively promoted the development and progress of human society, but it has also brought many environmental and safety problems. For example, many environmental or geotechnical problems in the waste rock and tailings are produced in ore mining and processing and the void left by mining [1]. One way to solve this problem is to backfill the void with waste rocks and tailings, supporting the ground and decreasing the management on the mine-site surface [2,3,4]. The backfilling method can directly transport waste rocks and tailings to the void or transport the waste rock and tailings to the void after adding water or cementitious materials [5]. It is called cemented paste backfill (CPB) mining method to backfill the void after mixing the tailings with cementitious material. Cemented paste backfill mainly consists of 70–85% (by solid weight) tailings discharged by the mineral processing plant, 3–7 wt% binder, and a certain amount of water [6]. Compared to other mining methods, it performs better in preventing surface subsidence and controlling ground pressure activities. It has a higher ore recovery rate, which reduces the accumulation of mine wastes on the surface, releases a large amount of ground space, and reduces the mining industry’s impact on the environment [7]. Since the first underground CPB was implemented at the Bad Grund Mine in Germany in 1979, which employed the fine mill rejects aggregate and silty filtered tailings as its main components [8], it is still widely used worldwide. The strength of backfilling body is an important index to evaluate the quality of CPB. Moreover, it is a crucial factor affecting the safety production of adjacent stope [9]. If the backfill strength is insufficient, safety accidents will quickly occur, and the environment will be polluted. At present, in-situ backfill strength is obtained by conducting uniaxial compression tests on backfill core samples, while it is time-consuming and the integrity of samples cannot be guaranteed [10].

The ultrasonic wave propagates as a bulk wave in an infinite medium, while the guided wave is confined to propagate in a medium due to the medium boundary, and the propagation direction is parallel to the medium boundary. The most significant difference between bulk and guided wave propagation is that guided wave propagation needs the boundary’s ‘guidance’. A large number of reflections, refractions, and modal transformations occur when acoustic waves propagate to the boundary of the waveguide structure, resulting in mutual interference between acoustic waves and continuous superposition, forming stable guided waves in a waveguide structure. The formation process of guided waves determines that the guided wave propagation has high sensitivity to defect characteristics and mechanical boundary changes. Based on this feature, guided wave detection technology had been formed [11]. Guided wave technology is a popular and relatively novel technology in the field of nondestructive testing. Compared with the traditional ultrasonic wave test, it has the advantages of long propagation distance, high detection efficiency, and wide detection range. However, the application of guided waves has only gradually matured in the past 20 years. Cawley et al. [12,13,14,15,16,17] conducted in-depth research on the propagation of guided waves in plates and pipelines and first applied it to the field of nondestructive testing. Simonetti et al. [18] studied the corrosion of pipelines by guided ultrasonic wave tomography. He C, Liu Z, et al. [19,20,21,22] proposed a series of methods for the nondestructive testing of steel strands and wind blades by ultrasonic guided waves [23,24,25]; meanwhile, they have developed a pipeline guided wave testing system, which is based on a time-reversal defect-identifying method and time-space focus theory [26,27,28,29]. D. Zou and Wang C et al. [30,31,32,33,34,35] conducted an in-depth study on the guided wave characteristics of the rock bolts. Li J and Zima B [36,37] explored the application of guided wave nondestructive testing technology to the integrity testing of reinforced concrete materials through theories and experiments. Literature review shows that guided waves have rarely been reported in the strength detection of mine backfill.

As the curing time goes on, the types and speciation of hydration reaction products inside the cemented paste backfill changes, which will cause the strength of CPB to differ [38] and affect the propagation of guided waves in cemented paste backfill mass. Then a variety of acoustic parameters such as wave velocity and attenuation during age means the change of backfilling mechanical properties. Therefore, the corresponding acoustic parameters of the guided wave to CPB strength, the guided wave monitoring method of strength development of cemented paste backfill mass can be realized.

In this paper, we try to find out the relationship between acoustic characteristics of guided waves propagating in CPB and the mechanical properties of CPB and propose a new method for detecting the strength of CPB using guided waves. The frequency and period of the excitation longitudinal guided waves were first optimized, and then the propagation velocity and attenuation changes of guided waves in CPBs at different cement-tailings ratios and different curing ages were tested. Combined with the uniaxial compression strength of CPB, a relation between CPB strength and the guided wave acoustic parameters was established.

## 2. Materials and Methods

### 2.1. Materials

**Figure 1:**
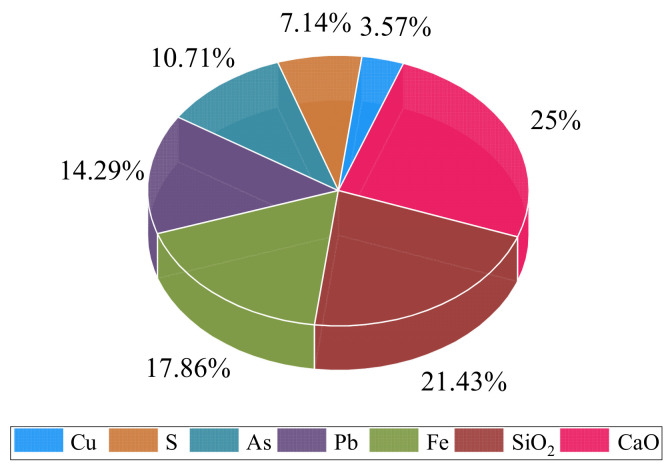
Main chemical properties of the tailings.

The unclassified tailings come from the tailings pond of a copper mine. The chemical composition of the tailings is shown in Figure 1. The main chemical compositions are SiO_2_, CaO, Fe, and S, which account for 71.43% of the tailings’ weight. The particle size distribution of tailings is listed in Table 1. The tailings have a specific gravity of 2.75 and an average moisture content of 1.17%. It can be seen from Table 1 that the tailings with a particle size larger than 0.074 mm account for 42.16%. Therefore, according to the commonly used classification standard [39], the whole tailings in the test are classified as coarse tailings.

No. 32.5 ordinary Portland cement was used as a binder agent, commonly used in the copper mine. Ordinary tap water was used to mix binder and tailings in this study.

### 2.2. Specimens for Test

#### 2.2.1. Background

Cemented paste backfill mining method is adopted in a copper mine, and the void is backfilled in two phases. The concentration of the first stage backfilling slurry is 70%, and the cement-tailings ratio is 1:4. When backfilling to 1/2 of an inlet section, the first stage backfilling is terminated, the second stage backfilling slurry concentration is 70%, but the cement-tailings ratio is 1:8. To be combined with the field, the concentration of test specimens prepared in this test is 70%, and cement-tailings ratios are 1:4 and 1:8, respectively, representing the first-stage and second-stage backfill.

#### 2.2.2. Specimens for Guided Wave Test

Generally, the waveguide structure, including a waveguide rod and several sensors, is used to excite and receive signals. Therefore, a waveguide structure was buried in the CPB specimen, with part of the waveguide structure exposed to the air.

**Figure 2:**
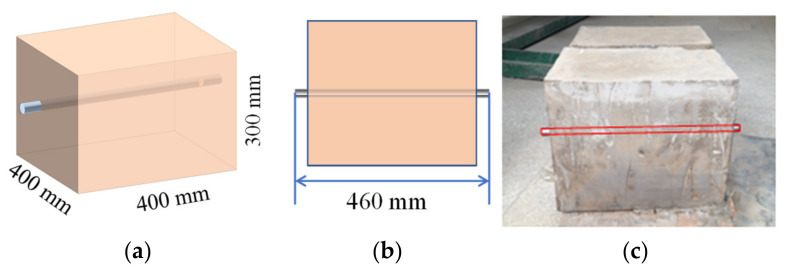
The dimension of specimens for guided wave testing. (**a**) Side view; (**b**) Top view; (**c**) Photograph.

The size of guided wave test pieces is 400 mm × 400 mm × 300 mm, the concentration of test specimens prepared in this test is 70%, and cement-tailings ratios are 1:4 and 1:8. With a diameter of 20 mm and length of 460 mm, a waveguide was buried in the longitudinal center of the piece, letting 30 mm at each end of the waveguide rod fix the sensor. The dimension diagram of specimens is shown in Figure 2. For the convenience of explanation, the 1:4 CPB sample was numbered ‘A’, and the 1:8 CPB sample was numbered ‘B’.

Before preparing test pieces, we placed the waveguide rod in the prefabricated hole. The weighed materials were mixed and homogenized for about 10 min. Then, the stirred slurry was poured into the test mold to be vibrated evenly. Demoulding after curing for three days, and placed the test piece into an environmental chamber with the controlled temperature at 20 ± 2 °C and 90% ± 5% relative humidity.

#### 2.2.3. Specimens for Uniaxial Compression Strength (UCS) Test

In this section, specimens for UCS tests are prepared with a concentration of 70%, and the cement-tailings ratio is 1:4 and 1:8, respectively, and the curing time is 3, 7, 14, and 28 days. The tailings, binder, and water with different proportions were prepared by an electronic scale with an accuracy of 0.01 g and thoroughly homogenized for about 5 min to produce the desired CPB mixtures. Then the CPB slurry was molded to a 50 mm × 100 mm cylinder. Next, put the prepared test specimens into an environmental chamber with the controlled temperature at 20 ± 2 °C and 90% ± 5% relative humidity.

### 2.3. Guided Wave Test

During the consolidation process, the phase state and microstructure of CPB constantly change, making the acoustic characteristics of guided wave propagation change. In this experiment, the parameters of the guided wave are optimized. Firstly, the same frequency with different cycles (within eight cycles) of excitation waves was adopted to determine the optimal cycle, and then the same cycle with different frequencies (20–70 kHz) of excitation waves was used to find the best frequency. These tests were carried out on CPB with the ratio of 1:4 and 1:8 and the concentration of 70%. The monitoring time was from 1 d to 42 d. The solidification process of the specimen is changing from a fluid to an elastoplastic body, and the change of this process will definitely lead to the change of the mechanical parameters of the specimen. The wave velocity and attenuation characteristics are obtained through the guided wave test to reflect the solidification process of the filling. In this way, the relationship between the strength of the filled body and the wave velocity and attenuation is established. 

#### 2.3.1. Test Set-Up and Procedure

**Figure 3:**
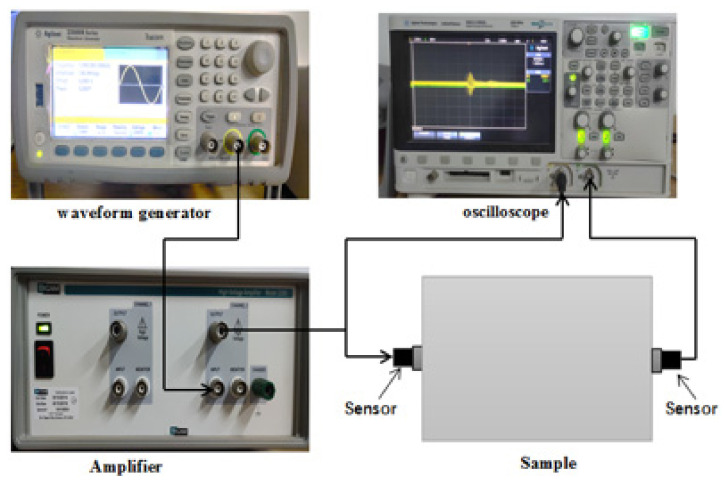
Diagram of guided wave testing system.

Guided wave testing system includes waveform generator, oscilloscope, signal power amplifier, sensors, et al. Connect as shown in Figure 3. Firstly, sensors were connected with the waveguide rod. Then, a thin layer of coupling agent was coated on the contact surfaces of the sensor and waveguide rod to ensure complete contact. Only one channel was used for the waveform generator after turning on the signal waveform generator, preheated for 10 min. The waveform generator generated excitation waves by reading the waveform file. After amplifying by the power amplifier, excitation wave entered the waveguide rod through the sensor. Finally, the received signal was denoised by the signal acquisition system and then stored in a U disk.

The excitation wave in this test comes from the following signal function Equation (1):

(1) Asin(2πft)×(1−cos(2πft/n))

where *A* is amplitude, *f* is the frequency, *t* is the duration, and *n* is the number of input cycles. The larger *n* is, the higher the energy of the excitation wave and the easier the resulting reflected wave is to identify. However, increasing *n* also increases the width of the wave packet, resulting in the overlap of the wave packet of the excitation wave with the wave packet of the reflection wave. Therefore, *n* cannot be too large or too small.

#### 2.3.2. Guided Wave Velocity and Attenuation Data Processing

The collected data need to be processed to obtain the desired guided wave acoustic parameters, wherein the wave velocity of the guided wave is calculated by the following Equation (2):

(2) $$V=\frac{L}{\Delta t}$$

where *V* represents wave velocity, *L* represents the waveguide length, ∆*t* represents the time difference between the original signal and the received signal.

**Figure 4:**
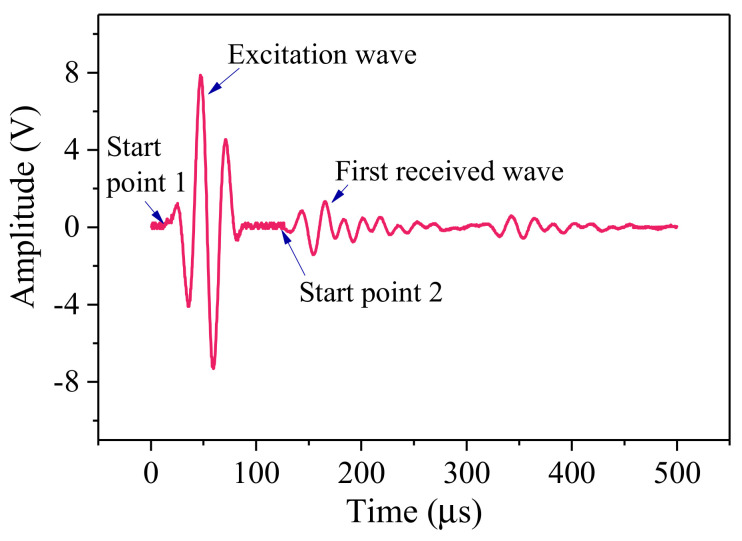
Pickup at the starting point and the time difference between the excitation and reception waves.

To reduce the error, the time difference of data processing takes a value between the starting point of the original signal and the first received wave, as shown in Figure 4. The first vibration change of the signal waveform is the starting point, and the starting point of the excitation and reception waves is specifically the point where the amplitude of the excitation and reception waves cross the threshold for the first time.

In addition to the velocity of guided waves, the attenuation of guided waves can also reflect the change of cemented paste backfill strength. Therefore, the attenuation value *A_t_* is calculated by the following Equation (3):

(3) $$A_{t}=-\frac{20}{L}\mathrm{lg}(\frac{P}{P_{ref}})$$

where *P_ref_* represents the peak-to-peak value of the excitation wave, *P* represents the peak-to-peak value of the wave captured from the other end of the waveguide, *L* is the length of the waveguide.

### 2.4. UCS Test

**Figure 5:**
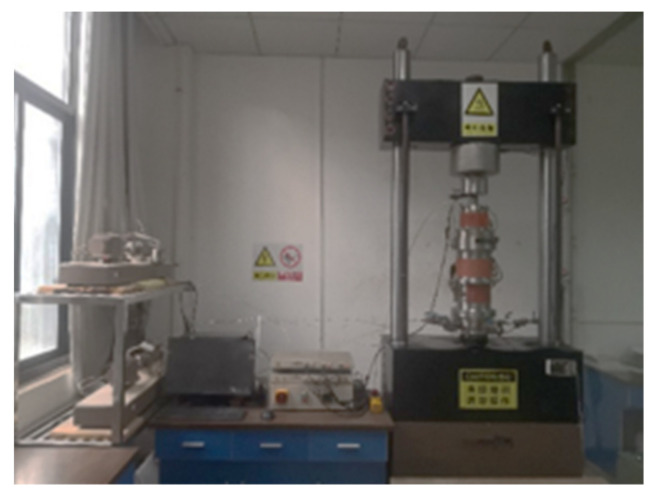
The High-pressure triaxial testing system.

Uniaxial compressive strength is one of the important indexes to evaluate the quality of backfill. The High-pressure triaxial testing system produced by British GDS Instruments was used in this test, as shown in Figure 5. In the test progressing, specimens were loaded under a constant force ratio of 0.5 kN/s. To avoid random errors in the tests, referring to the test standard ASTMC 39, four specimens were taken for each group of tests.

## 3. Results and Discussion

### 3.1. Optimization of Excitation Wave

To obtain the effective acoustic parameters of guided waves, it is necessary to select the optimal parameters of the excitation wave. The main parameters of the excitation wave are cycle and frequency. In this section, the appropriate cycle and frequency of the excitation wave were selected by analyzing the following two parts.

#### 3.1.1. Optimization of the Excitation Wave Cycle

**Figure 6:**
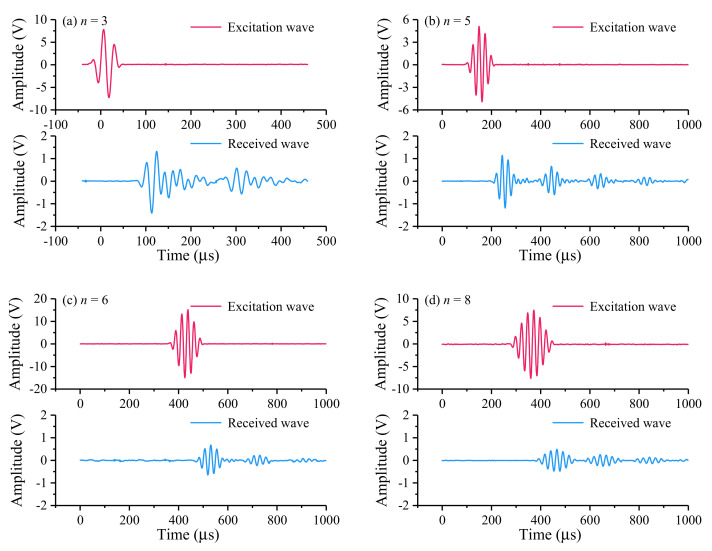
Effect of excitation wave parameter *n* on the receiving wave. The characteristics of the received wave are observed by changing the excitation wave parameter *n*. The amplitude decay, wave packet shape and overlap of the received wave are compared to optimize the parameter *n*.

The signal waveforms of the same frequency (within 100 kHz) in different cycles were used to test. Due to space constraints, following Figure 6 shows four representative signal waveforms under different cycles (*n* = 3, 5, 6, 8) at 40 kHz.

As shown in Figure 6, when the excitation waves with the same frequency but different periods were generated, the reflection formed by the guided wave propagating in the rod was different. The more period the excitation wave, the more incredible energy it will generate and the more pronounced the received wave can be formed, which is convenient to analyze. As shown in Figure 6a,b, it can be seen that the first end-reflected wave magnitude of 5 cycles has less attenuation than three cycles. The amplitude of 3 cycles was reduced from 15.09 v to 2.73 v, a decrease of 81.90%; the amplitude of 5 cycles was reduced from 10.00 v to 2.33 v, a decrease of 76.74%, indicating that the reflected wave with five cycles has more energy than that with three cycles, and the peaks of the excitation wave and the reflection wave with five cycles are more straightforward to distinguish than the waves with three cycles. Hence, it is more convenient to calculate the time difference between the peaks. However, increasing the number of cycles also increases the width of the wave packet, as shown in Figure 6c,d, which will cause the wave packet of the excitation wave to overlap with the reflected wave of the waveguide and cannot be identified. The amplitude of guided wave with six cycles reduced from 30.12 v to 1.32 v, with a decrease of 95.61%, and the amplitude of guided wave with eight cycles decreased from 15.11 v to 0.97 v, with a decrease of 93.60%. In conclusion, the excitation wave with five periods is the most suitable for this test.

#### 3.1.2. Optimization of the Excitation Wave Frequency

**Figure 7:**
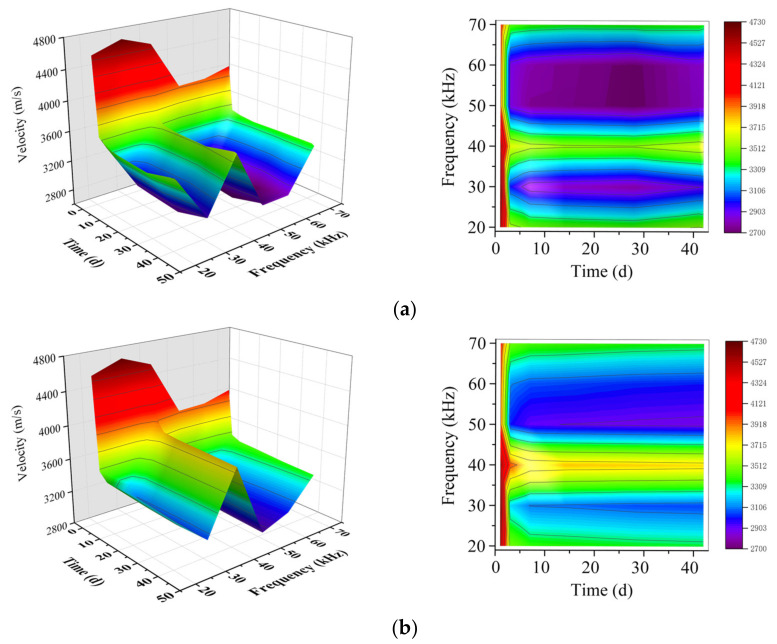
Velocities at different guided wave frequencies; (**a**) sample A; (**b**) sample B.

The same cycle with different frequencies (20–70 kHz) of the excitation waves was used to detect guided waves’ velocity and attenuation in CPBs. The results were illustrated in the following figures. Figure 7 shows the change of velocity with curing age at different guided wave frequencies. The result shows that the sensitivity of different frequency excitation wave to the detected structure is different. The overall trend of wave velocity changes at various frequencies decreases with the curing time, especially the initial wave velocity changes most apparent, and the wave velocity changes at the middle and late curing periods are small and tend to be stable. As shown in Figure 7, sample A has noticeable wave velocity variation at the excitation wave frequencies of 30 kHz, 50 kHz, and 60 kHz. The differences between the first day and the second day are all about 1000 m/s, among them, the wave velocity changes the most when the excitation frequency is 30 kHz, and the difference reaches 1364.33 m/s; For sample B, the wave velocity changes significantly when the excitation wave frequencies were 20 kHz, 30 kHz, and 50 kHz. At the frequency of 30 kHz, the maximum difference between the first day and the second day reaches 1300 m/s.

It can also be demonstrated from Figure 7 that the influence of CPB with a different cement-tailings ratio on wave velocity is different. From the stable value of wave velocity in the later curing period, velocity in sample A is lower than that in sample B.

**Figure 8:**
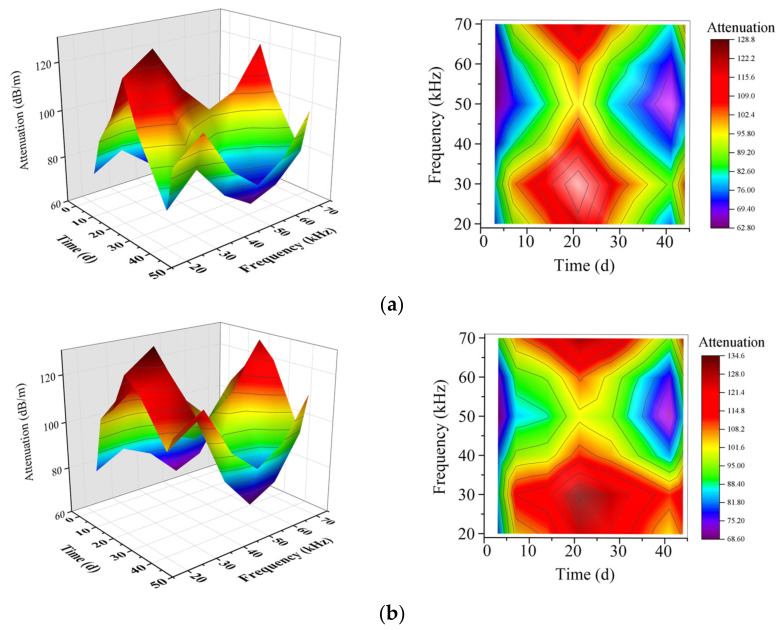
Attenuation at different guided wave frequencies; (**a**) sample A; (**b**) sample B.

Figure 8 shows the change of attenuation with curing age at different guided wave frequencies. It can be seen from the figure that according to the change of attenuation value with age, as the curing age increases, the attenuation change trend of guided waves in samples A and B is the same, which increases first and then decreases. From the initial curing to the age of 21 d, the attenuation shows an increasing trend, reaching the maximum value at 21 d and decreasing. When the excitation frequencies are 20 kHz, 30 kHz, and 70 kHz, the attenuation is more prominent, the maximum attenuation value of all three exceed 120 dB/m, and the guided wave attenuation is the smallest at 50 kHz. Among them, the attenuation value at 20 kHz and 70 kHz are relatively close, but the attenuation value of guided waves reaches the maximum at 30 kHz. The attenuation of guided waves in CPB with different cement-tailings ratios is also different. In the frequency range 20 kHz to 70 kHz, the attenuation value of specimens with a cement-tailings ratio of 1:8 is more significant than that with a cement-tailings ratio of 1:4. From the above results, it can be seen that the excitation wave with a frequency of 30 kHz is more sensitive to the detection of CPB, so the best frequency of the guided wave is 30 kHz. Therefore, combined with the conclusion of Section 3.1.1, a cycle of 5 and a frequency of 30 kHz are the most suitable parameters for monitoring CPB.

### 3.2. Relationship between UCS and Velocity of Guided Wave

When specimens were tested using guided waves, specimens with curing times of 3 d, 7 d, 14 d, and 28 d were conducted uniaxial compressive testing simultaneously. The test results are shown in Table 2.

As demonstrated in Table 2, the UCS of CPB regularly increases with curing time. From the results obtained in Section 3.1.2, the guided wave propagation in CPB also changes regularly with curing time. Based on this, the uniaxial compressive strength can be correlated with velocity or attenuation of guided waves in CPB under the corresponding curing time, and relationships between UCS and velocity or attenuation can be found. In engineering practice, the waveguide rod structure can be applied to monitor the strength of CPB, thus realizing the on-site monitoring of the strength of cemented paste backfill. The curve between the UCS and guided wave velocity of CPB was fitted in this section.

**Figure 9:**
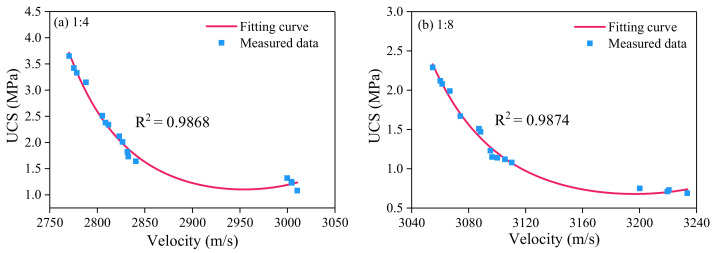
Relationship between UCS and velocity data for different cement-tailings ratio. Quantification of UCS by wave velocity.

Compared with various fitting schemes, an exponential function was finally selected for fitting, and the results are shown in Figure 9. The fitting of UCS and wave velocity of CPB with cement-tailings ratio 1:4 is illustrated in Figure 9a, and the fitting formula is shown in the following Equation (4), with a correlation coefficient (R2) of 0.9868. The fitting of UCS and wave velocity of CPB with cement-tailings ratio 1:8 is shown in Figure 9b, and the fitting formula is shown in the following Equation (5), with a correlation coefficient (R2) of 0.9874.

(4) $$y=e^{\left(313.9545-0.21249x+3.5965\times 10^{-5}x^{2}\right)}$$

(5) $$y=e^{\left(626.11828-0.39199x+6.1315\times 10^{-5}x^{2}\right)}$$

where *y* represents the UCS, and *x* represents the wave velocity of guided waves in CPB.

The correlation coefficients of fitting are all greater than 0.98, so the fitting is reliable. The strength changes of the two kinds of cement-tailings ratio CPB are the same with the change of wave velocity, and both show an exponential downward trend with the increase of wave velocity. The higher the wave velocity of guided waves propagating in cemented paste backfill mass, the lower the strength of CPB and the smaller change value of the strength.

### 3.3. Relationship between UCS and Guided Wave Attenuation

**Figure 10:**
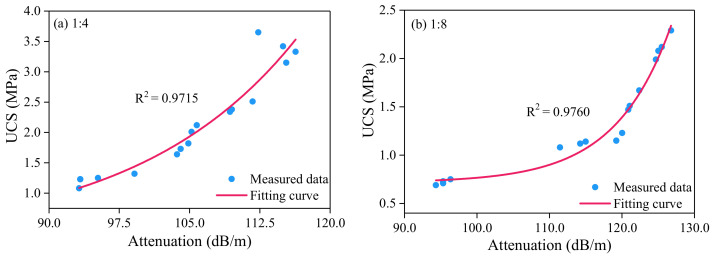
Relationship between UCS and attenuation data for different cement-tailings ratio. Quantification of UCS by wave attenuation.

This part fits the curve between UCS and the attenuation of guided waves in cemented paste backfill mass. Compared with various fitting schemes, the exponential function is still selected for fitting, and the results are shown in Figure 10.

The fitting of UCS and attenuation of guided wave in CPB with cement-tailings ratio 1:4 is shown in Figure 10a, and the fitting formula is shown in the following Equation (6), with a correlation coefficient (R2) of 0.9715. The fitting of UCS and attenuation of guided wave in CPB with cement-tailings ratio 1:8 is shown in Figure 10b, and the fitting formula is shown in the following Equation (7), with a correlation coefficient (R2) of 0.9760.

(6) $$y=0.22848+0.00372e^{0.05837x}$$

(7) $$y=0.71553+1.1902\times 10^{-7}e^{0.1296x}$$

where *y* represents the UCS, and *x* represents the attenuation of guided wave in CPB.

As is shown from the fitting function, the correlation coefficients of the fitting are both more than 0.97, and it is considered that the fitting is ideal. The strength variations of the two kinds of cement-tailings ratio are the same with the change of attenuation. UCS has the trend of the exponential increase with the increase of the wave velocity. The higher the attenuation of guided wave in CPB, the higher the UCS, and the greater the intensity variation value.

## 4. Conclusions

This section is not mandatory but can be added to the manuscript if the discussion is unusually long or complex.

(1) Guided waves with a frequency of 40 kHz and cycle numbers of 3, 5, 6, and 8 were used to detect cemented paste backfill samples. The results show that the larger cycle of the excitation wave, the easier the excitation wave and received wave overlap; they are difficult to distinguish. Combined with the attenuation of guided waves, the optimal detection cycle of the guided wave was determined to be 5.
(2) Guided waves with frequencies of 20–70 kHz were used to test samples A and B, respectively. The general trend of wave velocity at each frequency decreases with curing time and will finally be stable. However, with the increase of the curing period, the attenuation of guided waves in CPB increases first and then decreases. The test results indicate that both the velocity and attenuation of guided wave change most obviously at the frequency of 30 kHz. Therefore, 30 kHz is the optimal guided wave frequency for cemented paste backfill monitoring.
(3) Through the uniaxial compression test and guided wave test, the Mechanical and acoustic parameters of CPB were studied. The relationship between strength and wave velocity, strength and attenuation, and the corresponding exponential function fitting equations were found.

This study implicated that the guided wave technique as a nondestructive inspection method can also be used to evaluate CPB’s quality and properties besides defect detection. While some aspects still need to investigate the performance of CPB samples using the guided wave technique. In this paper, the guided wave testing uses the through-transmission method; whether the pulse-echo method still works remains determined. Furthermore, the influence of cement-tailings ratio on acoustic parameters has been studied, but the influence of slurry concentration and aggregate particle size on guided wave acoustic parameters still needs further study.

## Figures and Tables

**Table 1:** Particle size distribution of the tailings samples.

**Particle Size/um**	0~33	33~45	45~74	>74	
**Content/%**	38.82	4.31	14.71	42.16	100

**Table 2:** Uniaxial compressive strength of CPBs.

	UCS/MPa			
	3 d	7 d	14 d	28 d
Cement-tailings Ratio (1:4)	1.25	1.73	2.12	3.42
	1.23	2.01	2.15	3.33
	1.08	1.82	2.38	3.15
	1.32	1.64	2.34	3.65
Cement-tailings Ratio (1:8)	0.69	1.14	1.67	2.12
	0.73	1.12	1.47	2.08
	0.71	1.15	1.23	1.99
	0.75	1.08	1.51	2.29

## Data Availability

The data presented in this study are available on request from the corresponding author.
